# Metastatic Prostate Adenocarcinoma Mimicking Multifocal Periapical Pathosis

**DOI:** 10.7759/cureus.104778

**Published:** 2026-03-06

**Authors:** Pinar Emecen-Huja, Tina R Woods, Perry Stamatiades, Jacqueline Medina, Angela J Yoon, Dalton P Wilson, James Loe, Douglas Alterman, Jeffrey S Kirk

**Affiliations:** 1 Advanced Specialty Sciences, Medical University of South Carolina (MUSC) James B. Edwards College of Dental Medicine, Charleston, USA; 2 Biomedical and Community Health Sciences, Medical University of South Carolina (MUSC) James B. Edwards College of Dental Medicine, Charleston, USA; 3 Reconstructive and Rehabilitation Sciences, Medical University of South Carolina (MUSC) James B. Edwards College of Dental Medicine, Charleston, USA

**Keywords:** histopathologic examination, intraoral radiograph, metastatic prostate adenocarcinoma, periapical pathology, radiolucent lesions of jaw

## Abstract

The jaw is an uncommon site for metastasis, accounting for 1% of all oral cancers. The posterior mandible is the most common site of metastatic deposits, which typically present as ill-defined radiolucency. We present a case of metastatic prostate adenocarcinoma found during routine radiographic examination, resembling that of periapical pathology bilaterally in the mandible. The lesions presented as well-defined radiolucencies at the apex of non-vital teeth. Both lesions were revealed to be metastatic adenocarcinoma during histopathologic examination, emphasizing the importance of including malignant neoplasm in the differential diagnosis.

## Introduction

Metastatic carcinoma is the most common type of malignant neoplasm found in bone [1]. The primary sites that typically metastasize to bone include the breast, lung, thyroid, prostate, and kidney. Metastatic neoplasm mainly affects the vertebrae, ribs, pelvis, and skull [2].

The jaws and oral mucosa are rare sites for metastasis, with about 1% of oral malignancies attributable to metastatic deposits [3]. However, the incidence of metastasis to the jaw is suspected to be higher. Indeed, micrometastatic foci were found in the jaws in 16% of autopsies of patients without prior evidence on imaging studies [4]. Prompt detection is critical for proper diagnosis and intervention. Hence, metastatic neoplasm should be considered in the differential diagnosis when managing inflammatory or reactive lesions of the jaw [5-9].

We present a case of metastatic prostate adenocarcinoma observed as well-defined periapical radiolucency of the right and left mandible. While the periapical pathologies, including periapical granuloma and cyst, are the most likely diagnosis, it is vital to perform histopathologic examination of the lucent lesions. In our case, both lesions resembling periapical pathology were diagnosed as multifocal metastatic prostate cancer. Metastatic lesions presenting as multifocal periapical pathologies are rare, and accurate diagnosis and prompt management are critical to patient survival.

## Case presentation

A 75-year-old male was referred to the dental clinic for dental clearance in October 2024. The medical history was significant for type 2 diabetes mellitus, hyperlipidemia, hypertension, fatty liver, and carotid arteriosclerosis. The patient had elevated prostate-specific antigen (PSA) in October 2021 and was referred to a urologist for a biopsy, which revealed Gleason 10 prostate cancer. The positron emission tomography (PET) scan (performed by an outside hospital; images unavailable) demonstrated a hypermetabolic mass involving a large portion of the prostate, extending posteriorly through the layers of fascia into the rectum. Multiple prominent hypermetabolic pelvic and retroperitoneal lymph nodes were seen, consistent with metastatic nodal disease. There was no evidence of involvement of the pubic ramus. The diagnosis of castration-naïve Gleason 10 Stage Iva (T4M1) high-risk adenocarcinoma of the prostate was rendered. The patient initiated the androgen deprivation therapy (ADT) with Eligard and Casodex tablets, which was well-tolerated. After six months of treatment, the PSA level decreased from 3.0 (normal range: 0.1-4.0 ng/ml) to 0.5, demonstrating a positive response.

A year after initiation of the ADT treatment in January 2023, the PSA level rose to 3.0, concerning for castration-resistant disease. The Prostate-Specific Membrane Antigen (PSMA) PET scan (performed at an outside hospital; images unavailable) revealed locally advanced disease with nodal involvement and scattered areas of bone uptake, concerning for bone metastasis. The therapy was changed from Casodex to Apalutamide with a subsequent decrease in the PSA level to 2.5. In December 2023, the PSMA PET scan showed increased bone metastases involving the sacrum and iliac bone and a rapidly rising PSA. Due to progressive disease, the patient was placed on a second-line therapy of ADT combined with Taxotere. He experienced a minimal, short-lived response to treatment, and then PSA rose again rapidly. The patient began third-line therapy with cabazitaxel in April 2024, achieving a stable PSA by May 2024. Following cycle 1, the patient developed severe thrombocytopenia with platelets as low as 20,000. Bone marrow biopsy showed marrow fibrosis. Due to his severe cytopenia being a significant barrier to therapy, the patient was placed on abiraterone.

**Figure 1:**
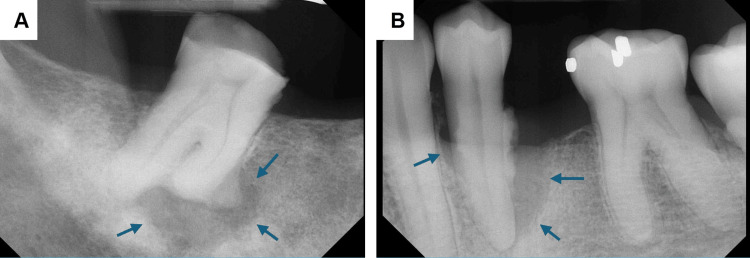
Radiograph showing (A) periapical lucency of tooth #31 and (B) radicular lucency of tooth #17 Blue arrows are pointing to the lucency associated with the teeth.

The PSMA PET scan in May 2024 showed progression of osseous disease and involvement of the liver. The CT-guided biopsy confirmed metastatic prostate adenocarcinoma of the liver. Prior to initiating Xgeva therapy, the patient was referred to the dental clinic for the removal of infected teeth. The clinical examination and intraoral radiograph revealed a non-restorable and non-vital left mandibular premolar and right mandibular molar with radicular and periapical radiolucency, respectively (Figure 1). The patient was asymptomatic without pain or paresthesia. There was no bone expansion. With a multifocal radiolucent presentation of the lesions, specifically associated with non-vital teeth without bone expansion, the clinical diagnosis was periapical pathology. Metastasis was also considered in the differential diagnosis, although malignant lesions tend to demonstrate locally aggressive clinical behavior with bone expansion and ill-defined margins on radiographs.

**Figure 2:**
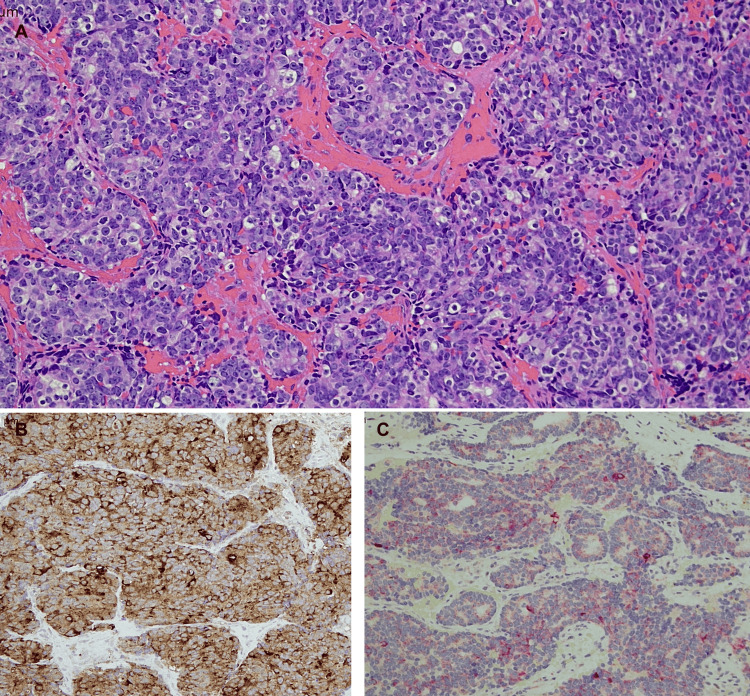
Photomicrographs of metastatic prostate adenocarcinoma: (A) H&E (200×) showing diffuse proliferation of malignant cells, (B) positive PSA stain (200×), and (C) PIN-4 stain (200×) with positive P504S red cytoplasmic staining and a lack of basal cell p63+HMWK brown staining

Teeth were extracted, and the radiolucent areas were curetted. The histologic examination of the tissue revealed a diffuse malignant proliferation of round basophilic and hyperchromatic cells with basophilic cytoplasm and prominent nuclei, arranged in nests and individual cells (Figure 2). Immunohistochemical stains were positive for PSA and negative for synaptophysin (neuroendocrine). The prostate triple stain (PIN-4) demonstrated P504S positivity in the cytoplasm of tumor cells, with a lack of basal cell staining (p63 and HMWK negative). PSA positivity confirms the prostate origin, while the P504S positivity and p63 negativity are the standard for differentiating benign from malignant adenocarcinoma. The patient received radiotherapy. As of June 2025, the patient is continuing with the chemotherapy for prostate adenocarcinoma.

## Discussion

Metastatic jaw lesions are most common among middle-aged and elderly patients, with a significantly higher frequency in those over 50 years of age [10-12]. Oral metastasis is commonly found in the jaw, especially in the mandible [13]. There is a predilection for the posterior mandible because of its rich blood supply in active areas of hematopoiesis [8]. The gingiva or mucosa of the alveolar ridge is the most common soft-tissue site [5,8]. A study by Daley et al. reported eight cases of metastatic prostate carcinoma: five cases to the mandible, one to the maxilla, one to the palate, and two to soft tissues (maxillary alveolar mucosa and buccal vestibule) [14]. Recent reports of metastatic prostate adenocarcinoma to the oral cavity are listed in Table 1 [15-18].

**Table 1:** Metastatic prostate adenocarcinoma to the oral cavity

Age	Site	Symptoms	Diagnostic Method	Clinical Outcome	Reference
76	Posterior Mandible	Bone expansion	Imaging/Histopathology	In remission at year 7	15
64	Mandible	Bone expansion, cortical perforation, and paresthesia of the ipsilateral lip	Imaging/Histopathology	In remission	16
79	Posterior Mandible	Bone expansion, tenderness, and pain while eating	Imaging/Histopathology	Not available	17
60	Mandible	Asymptomatic bone expansion, draining sinus, ipsilateral lip paresthesia	Imaging/Histopathology	Not available	18

In the presented case, the metastatic lesion presented as an incidental radiographic finding in an asymptomatic patient. The clinical oral examination typically reveals swelling with tenderness over the affected area due to underlying bone expansion [1,2]. Radiographically, metastatic neoplasms commonly appear as lucency with ill-defined margins, simulating osteomyelitis [2]. If extensive bone resorption is present, a pathologic fracture may be present. Metastatic prostate carcinoma may produce calcifications [2]. Subsequently, approximately 50% present as a mixed opaque-lucent lesion, while 40% present as a radiolucency [7,9]. In ~5% of cases, there may not be radiographic evidence of pathology [8]. Metastatic disease to the jaws may breach the cortical plate and invade the soft tissues, mimicking a mucosal infection [10]. As per standard medical practice, all lesions must be submitted for histologic examination for a definitive diagnosis and to rule out neoplasm, as in our case.

The differential diagnosis for a well-defined unilocular radiolucency at the apex of a non-vital tooth includes periapical granuloma, periapical cyst, and periapical scar. Odontogenic keratocyte and ameloblastoma, which can present as well- or ill-defined, uni- or multi-locular radiolucency mimicking other lucent lesions, are also included in the differential diagnoses. Hence, it is critical to include metastasis in the differential diagnosis, especially in the presence of primary carcinoma.

## Conclusions

While metastasis of malignant neoplasm to the jaw is uncommon, it is crucial to thoroughly curettage the lesion for histopathologic evaluation. While the prognosis is poor, with most patients surviving less than a year after jaw metastasis, proper treatment, such as radiotherapy, may improve survival.
